# A forest firefighting task area division method based on the SW-DBSCAN algorithm

**DOI:** 10.1038/s41598-026-42407-0

**Published:** 2026-03-18

**Authors:** Qiong Huang, Yu Huang

**Affiliations:** 1 Basic Courses Department, China Fire and Rescue Institute, Beijing, 102202 China; 2 Department of Emergency Communication and Information Engineering, China Fire and Rescue Institute, Beijing, 102202 China

**Keywords:** Forest fires, Task division, SW-DBSCAN, Sliding window, Engineering, Mathematics and computing

## Abstract

A new method is proposed for generating forest fire-fighting tasks using an improved Sliding Window-based Weighted Density-Based Spatial Clustering of Applications with Noise (SW-DBSCAN). To reduce the neighborhood search range of core objects and improve the clustering accuracy of key protected object, fire head, fire tail, and fire flank on the fire line, a sliding window is introduced, and a weighted similarity distance measurement method is designed in the DBSCAN framework. At the same time, evaluation indicators are proposed to evaluate the effectiveness of this method. Finally, several numerical simulations show that this method is feasible and flexible for generating forest firefighting tasks.

## Introduction

Forest fires are highly destructive. They not only damage forest ecosystems, but also threaten the lives and property of surrounding residents, causing incalculable losses. Effective forest firefighting operations are the most important. Timely and efficient fire suppression actions can minimize losses, protect ecological functions of the forest and the lives and property of surrounding residents. Forest firefighting task area division is complex and important in forest fire-fighting work. Based on the scientific analysis of fire environment, a fire line is delineated and several task areas are generated. The fire threat level, topography, and meteorological environment in each task area are similar. Accurate allocation of firefighting resources, precise deployment of rescue tactics, and reasonable overall planning will be carried out based on the fire conditions in each task area, thereby effectively improving the firefighting efficiency and reducing casualties and property losses. Therefore, scientific division of forest firefighting task areas is of great significance for improving the overall rescue.

Most scholars focus on optimizing helicopter firefighting task scheduling^1–3^ and planning unmanned aerial vehicle (UAV) firefighting tasks^4–6^. Xu et al. proposed a two-stage dynamic path planning model for the helicopter single-stage scheduling route planning problem^7^. The Ant Colony Optimization (IACO) algorithm was improved to solve the model, providing a reliable framework for forest fire rescue. Considering the threat of environmental factors in multiple forest fires, Shao et al. improved the optimization algorithms, and established a multi-UAV task allocation and path planning model^8^. This provides an effective solution for multi-UAV firefighting task planning in forest fires. Based on the clustering algorithm, a model is established for the task area division. The similarity characteristics of the fire behavior, topography, meteorology, and vegetation environment for all points on the fire line are classified.

Clustering divides the unlabeled datasets into several clusters based on the distribution characteristics and correlations of the data^9,10^, ensuring the maximum similarity within each cluster and the maximum dissimilarity between clusters. It includes partition-based clustering algorithms^11,12^, graph-based clustering algorithms^13,14^, hierarchy-based clustering algorithms^15,16^, and density-based clustering algorithms^17–20^. Partition-based clustering algorithms^21,22^ require specifying the number of categories before partitioning, and the random initial partition of data will affect the stability of clustering. Graph-based clustering algorithms represent the data as a graph structure for clustering^23,24^, and hierarchy-based clustering organizes the data into a tree structure to achieve clustering^25,26^. Both can handle clusters of arbitrary shapes shape without relying on the selection of initial centers. However, they have high computational complexity and are sensitive to noise. Density-based spatial clustering of applications with noise (DBSCAN) is a well-known unsupervised clustering algorithm that can identify clusters of arbitrary shapes and effectively filter noise in data^20,27,28^. It has wide applications in time-series^29–31^ and multi-modal data mining^16,18,32^. However, when the dataset is large, they will have long convergence time and high computation. The DBSCAN algorithm has been improved based on proximity graphs^33,34^, k-d trees^35,36^, and ball trees^37,38^. It can ensure high clustering accuracy while accelerating the computation, but increase the additional space complexity.

The task division in forest firefighting is to divide the fire line into several continuous sub-task areas. According to the principles of forest fire-fighting, priority should be given to extinguishing forest fires in key protection areas. At the same time, the operational plans for key protected objects, fire head, fire tail, and fire flank at the fire area are slightly different. Therefore, in clustering, it is essential to ensure that the clustering results are continuous on the fire line and can effectively distinguish key protection objects, fire heads, fire tails, and fire flanks. The DBSCAN algorithm clusters all data with the same density. The similarity distance metrics mainly include Euclidean distance^39,40^, Mahalanobis distance^41,42^, and Manhattan distance^43,44^. It can correctly divide clusters, but it is difficult to ensure the spatial continuity of clustering results and effectively identify key protected objects, fire head, fire tail, and fire flank on the fire line. Therefore, a Weighted DBSCAN algorithm based on a sliding window (SW-DBSCAN) is proposed. Through searching for density-reachable points in the sliding window of core objects and weighting key parts during the calculation of similarity distance metrics, effective division of forest fire-fighting tasks is achieved. The experimental results show that compared with the traditional DBSCAN algorithm, the SW-DBSCAN algorithm has high clustering effect, high efficiency, and high stability. Although the outlier rate has slightly increased, it is basically controlled in an acceptable range. This provides a more reliable basis for optimizing resource scheduling and tactical execution efficiency in rescue.

The main contributions of this work are drawn as follows:

1. Based on the basic principles of forest fire disposal and operations, the characteristics of forest firefighting task division are analyzed, and a mathematical model is established to solve the problem of forest fire-fighting task division;
2. Based on the objectives of forest firefighting task division, a DBSCAN algorithm with a sliding window is proposed. The sliding window significantly reduces the search range for directly density-reachable points of core objects, ensuring the continuity of forest firefighting tasks. The calculation onthe local density of the core object can effectively improve the execution efficiency of the algorithm;
3. A weighted similarity distance metric method is designed to increase the distance between key protected object, fire head, fire tail, and fire flank on the fire line for effective differentiation and better clustering;
4. A series of new evaluation indicators are designed to address the problems of insufficient evaluation indicators and unreasonable evaluation of experimental results in current clustering algorithms. Considering the continuity and recognizability of forest firefighting task area division, these indicators can better evaluate the effectiveness of the algorithm.

The structure is organized as follows: Sect. Methodology introduces the sources and preprocessing of data collection and the method for dividing the forest fire prevention task area. It includes the description and modeling of problems, the core idea and algorithm flow of the DBSCAN algorithm, and specific implementation of the improved algorithm, SW-DBSCAN; Sect. Experimental analysis compares the SW-DBSCAN with other algorithms through experiments, and Sect. Conclusions gives the main results and conclusions.

All custom code and scripts used in this study are archived in Zenodo with a persistent 10.5281/zenodo.18618461. The repository includes full documentation of code usage, dependencies, and reproducibility steps.

## Methodology

### Data source and preprocessing

**Fig. 1:**
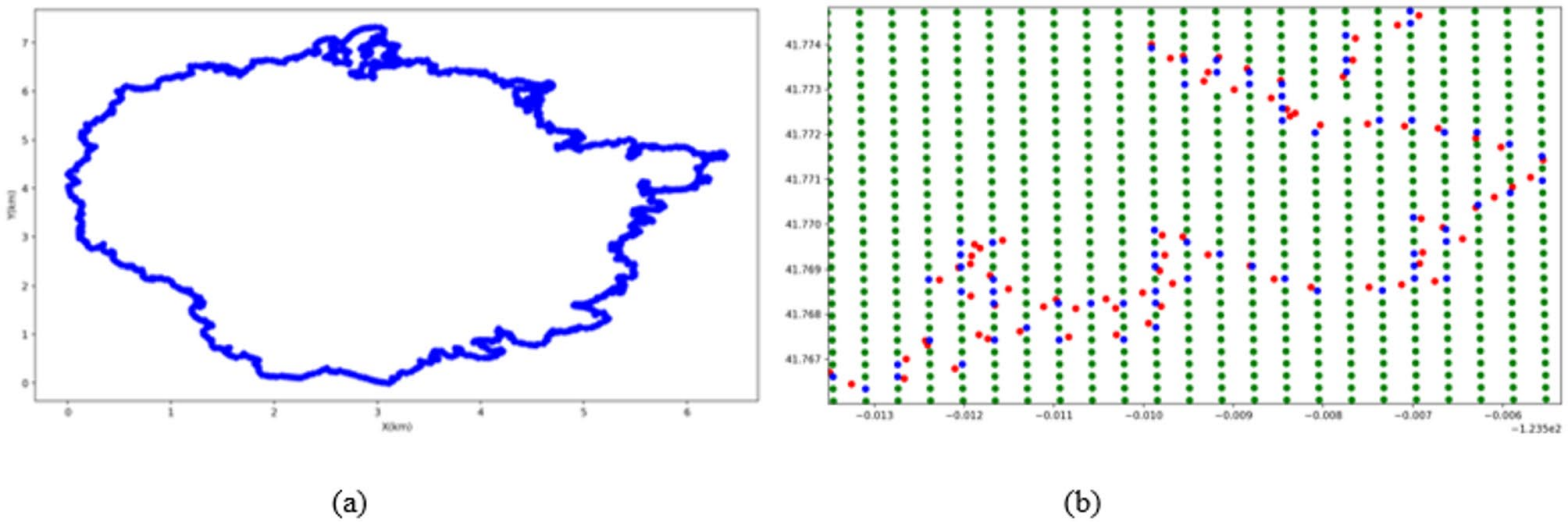
Data preprocessing.

The study area is located in the Oak Knoll region of Napa County, California, USA (38°21′30″N, 122°20′05″W). The experimental data were obtained through the FlamMap fire simulator^45^. To ensure sufficient data, 10 fire lines with a total length of about 282 km were extracted. Figure 1a shows the fire line at a certain moment when the fire occurred. The component values of the feature vectors at the fire line points can be sequentially obtained by organizing the input and output data of the simulator. Wind direction, wind speed, fire intensity, slope aspect, slope gradient, forest canopy density, and fuel type can be obtained by searching the corresponding values in the environmental and output files of the fire simulation that are closest to the fire line points. As shown in Fig. 1b, the red dots represent the positions of fire line points, and the blue dots represent the coordinate points of the grid cell where the fire line points are located, with a grid accuracy of 30 m. Fire head spread the fastest and has the strongest fire intensity, and the opposite is the fire tail. The parts on both sides between the fire head and fire tail are the fire flank. Based on the propagation speed of the fire line points and the relationship between propagation and wind directions, the types of key parts can be quantified in order as 1, 2, and 3 (corresponding to fire tail, fire wing, and fire head), respectively. Based on the relationship between the spread direction of the fire line point and the slope of that point, uphill fires are quantified as 1 or 2 (uphill fire or downhill fire). Depending on whether a key protection object is randomly generated on the fire line, it is assigned a value of 2, and all non-key objects are assigned a value of 1. To improve the convergence speed of the algorithm and eliminate the influence of different dimensions on data analysis, each component value is reduced to the interval (0, 1].

### Method

#### Problem description

According to the basic principles of forest fire disposal and operations, the division of forest firefighting tasks needs to consider multiple factors such as fire behavior, topography, and meteorology in the fire field. The continuous fire line areas under similar conditions should be divided into a firefighting sub-task area. According to the Regulations on Forest Fire Prevention^46^, key protected areas include residential areas, high-value forest areas, cultural heritage buildings and other important facilities. According to the normal definition^47^, fire head is the main direction of the fire development, referring to the part of the fire that spreads the fastest along the wind direction. Fire tail refers to the part of the fire that spreads the slowest upwind, and fire wing is located between the head and tail of the fire and spreads to both sides. In addition, the clustering results must be continuous on the fire line. The key protected objects must be effectively distinguished from the fire head, fire tail, and fire wing. This is because the operational plans of the key protected object, fire head, fire tail, and fire wing may be slightly different in the fire field.

In this study, a continuous fire line is discretized into $m$ points,$L=\left\{{l}_{i}\right|i=\mathrm{1,2},\ldots, m\}$,. The 10-dimensional feature vector of each fire line point is recorded as ${a}_{i}=(Ka;Kp;Wd;Ws;Fi;Ap;Sl;Cp;Fm;Ud)$, where $Ka,Kp,Wd,W$*s*$,Fi,Ap,Sl,Cp,Fm,Ud$ represent whether a point on the fire line is a key protection object, part types (fire head, fire tail, and fire wing), wind direction, wind speed, fire intensity, slope aspect, slope gradient, forest canopy density, fuel type, and whether it is an uphill fire. In addition, a sample set of the fire line $D=\{{a}_{1},{a}_{2},\ldots, {a}_{m}\}$ is established and divided into k disjoint clusters $\left\{{C}_{k}\right|k=\mathrm{1,2},\ldots, n\}$, and ${C_k}{ \cap _{k^{\prime} \ne k}}{C_{k^{\prime}}}=\emptyset$ and $D={\cup}_{k=1}^{n}{C}_{k}$. The following two conditions should be satisfied:

(1) Continuity: If the feature vector of the fire line point ${l}_{{j}^{{\prime}}}$ is ${a}_{{j}^{{\prime}}}\in{C}_{k}$, the feature vector of the fire line point ${l}_{j}$ is ${a}_{j}\in{C}_{k}$, and $j^{\prime} \ne j$, then fire line points ${l}_{{j}^{{\prime}}}$ and ${l}_{j}$ should be in a certain continuous interval on the fire line, ${l}_{{j}^{{\prime}}},{l}_{j}\in[{l}_{s},{l}_{e}],s,e\in\{\mathrm{1,2},..m\}$.

(2) Recognizability:

(i) If the $Ka$ dimension of the feature vector ${a}_{j}$ corresponding to the fireline point ${l}_{j}$ is denoted as ${a}_{j}^{Ka}$, ${a}_{j}\in{C}_{k}$, then there is no ${j}^{{\prime}}$, and ${a}_{{j}^{{\prime}}}^{Ka}\ne{a}_{j}^{Ka}$ and ${a}_{{j}^{{\prime}}}\in{C}_{k}$.
(ii) If the fire line point ${l}_{j}$ is a non-key protection object, the $Kp$ dimension of the feature vector ${a}_{j}$ corresponding to it is denoted as ${a}_{j}^{Kp}$, ${a}_{j}\in{C}_{k}$, then for any ${j}^{{\prime}}$, ${a}_{{j}^{{\prime}}}\in{C}_{k}$, and ${a}_{{j}^{{\prime}}}^{Kp}={a}_{j}^{Kp}$.

#### Improved DBSCAN algorithm

**Fig. 2:**
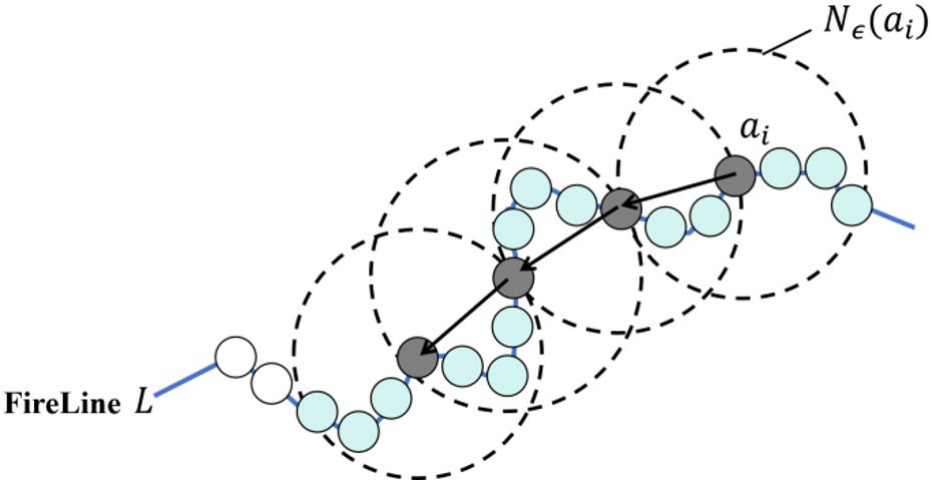
Density-reachability of DBSCAN algorithm.

**Fig. 3:**
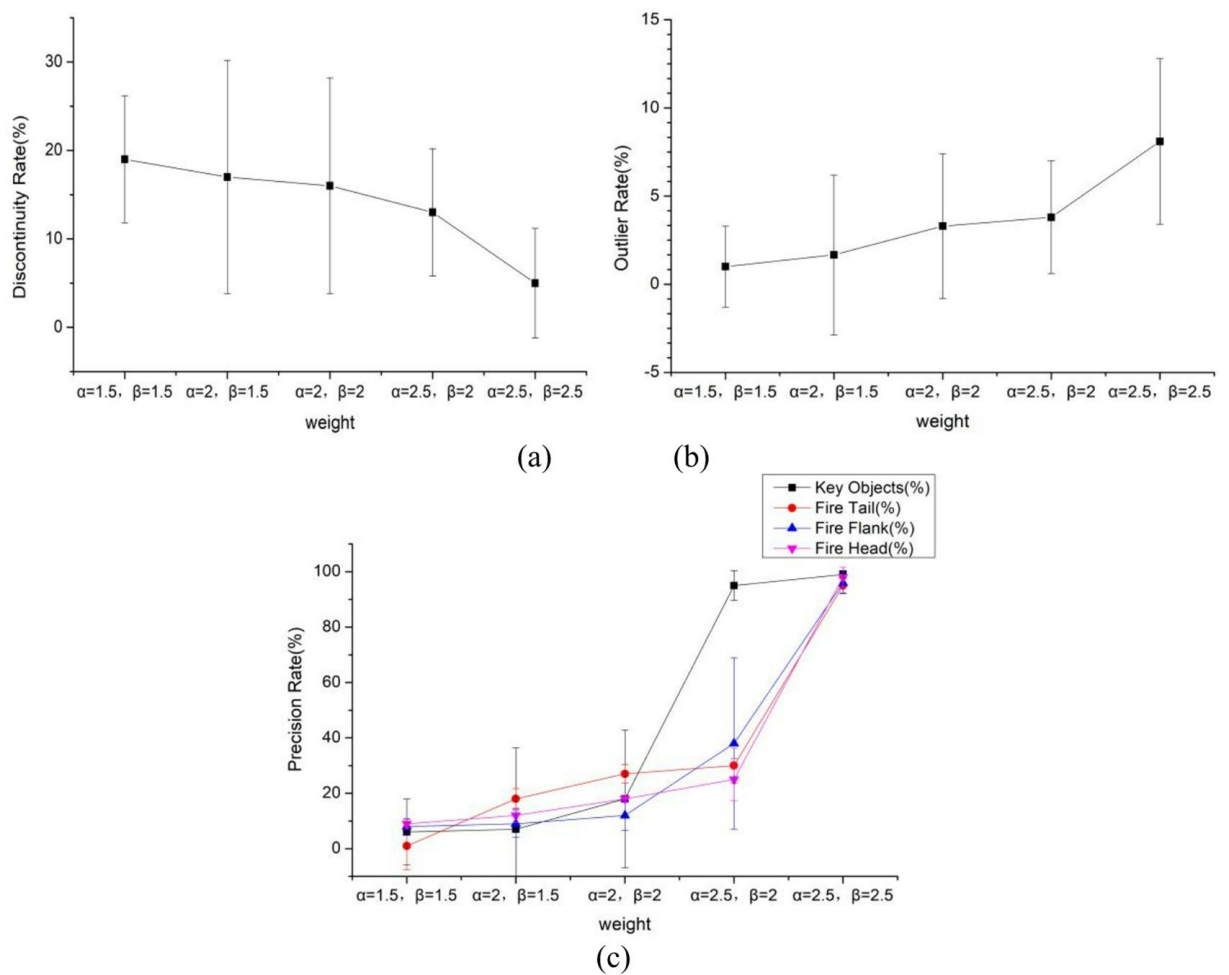
Influence of weights ${\upalpha}$ and ${\upbeta}$ on the algorithm.

DBSCAN (Density-Based Spatial Clustering of Applications with Noise) is a density-based clustering algorithm. It continuously expands the clusters based on the local connectivity of samples in the neighborhood to obtain the final clustering results. It does not require specifying the number of clusters in advance and can effectively handle clusters of arbitrary shapes with good robustness. The core idea of the DBSCAN algorithm is that if the neighborhood radius $\epsilon$ of a sample ${a}_{i}$ starting from a certain point on the fire line $L$, ${N_\epsilon }\left( {{a_i}} \right)=\left\{ {{a_j} \in D|dis{t_{ij}}\left( {{a_i},{a_j}} \right) \leqslant \epsilon } \right\}$, contains at least $MinPts$ samples, the sample is considered a core object. As shown in the gray dotted line point in Fig. 3, the region is a high-density area that can be marked as the same cluster. At the same time, the cluster is expanded through density-reachability. As shown in Fig. 2, the hot wire point extends in the direction indicated by the arrow.

The DBSCAN algorithm does not require a prior specified number of clusters and can effectively determine clusters of any shape based on the density of sample distribution with good robustness. However, since DBSCAN needs to calculate the distance between all sample pairs when searching for neighborhoods, its time complexity is $O\left({m}^{2}\right)$ (where $m$ is the number of samples) in the worst case, resulting in a long clustering convergence time for large sample sets. Although DBSCAN algorithm has been improved for this issue, for instance by using kd-tree^35,36^ for neighboring point search to identify the neighborhoods of all samples, and reducing the time complexity to $O\left(m{\mathrm{log}}_{2}m\right)$, it will cost more spatial complexity. In addition, the neighborhood search range of core objects in the DBSCAN algorithm is the entire dataset. It is easy to divide the core objects and distant non-continuous sample points with the same density into a cluster, which cannot meet the continuity requirement of forest fire-fighting task area division. At the same time, the similarity distance measurement of the DBSCAN algorithm only focuses on the real distance between samples, marking nearby samples as the same cluster. This makes it difficult to accurately distinguish key protected object, fire head, fire tail, and fire flank on the fire line. To this end, the SW-DBSCAN algorithm is proposed. When calculating the distance of the sample points, the weight coefficients ${\upalpha}$and ${\upbeta}({\upalpha}>1, {\upbeta}>1)$ are added respectively to the dimensions of key protected objects and part types in the sample feature vector, so as to increase the distance between different key parts on the fire line. The distance between the same key parts will not be affected, achieving identification. In addition, during the clustering process, for each sample, only the distance between this sample and the samples within the sliding window starting from it is calculated to improve the execution efficiency of the algorithm. The direct density reachable point search range of each sample is limited in a sliding window, effectively solving the continuity problem.

##### Related definitions of SW-DBSCAN

Relevant definitions in the SW-DBSCAN algorithm are as follows:

###### Definition 1

Weighted distance: Let ${a}_{i}^{Ka}$ and ${a}_{i}^{Kp}$ be $Ka$ and $Kp$ for sample ${a_i}$(${a_i} \in D$) respectively, ${a}_{i}^{k}(k=2,..9)$ represents the remaining characteristics of the sample ${a_i}$, ${\mathrm{\boldsymbol{\upalpha}}}>1$ and ${\mathrm{\boldsymbol{\upbeta}}}>1$. The distance between samples ${a}_{i}$ and ${a_j}$(${a_j} \in D$) is defined as${dist}_{weighted}({a}_{i},{a}_{j})=\sqrt{{{\upalpha}\bullet({a}_{i}^{ka}-{a}_{j}^{ka})}^{2}+{\upbeta}\bullet{({a}_{i}^{kp}-{a}_{j}^{kp})}^{2}+\sum_{k=2}^{9}{({a}_{i}^{k}-{a}_{j}^{k})}^{2}}$, denoted as ${dist\_w}_{i,j}$.

###### Definition 2

Weighted distance matrix: A matrix containing the weighted distances between each pair of samples ${a_i}$(${a_i} \in D$) an ${a_j}$(${a_j} \in D$),

$${DIST}_{weighted}=\left[\begin{array}{ccc}\begin{array}{ccc}0&{dist\_w}_{\mathrm{1,2}}&{dist\_w}_{\mathrm{1,3}}\\0&0&{dist\_w}_{\mathrm{2,3}}\end{array}&\begin{array}{ccc}\dots&{dist\_w}_{1,d-1}&0\\{dist\_w}_{\mathrm{2,4}}&\dots&{dist\_w}_{2,d}\end{array}&\begin{array}{ccc}0&\dots&0\\0&\dots&0\end{array}\\ \vdots& \vdots& \vdots \\\begin{array}{c}\begin{array}{ccc}0&0&\dots\\{dist\_w}_{m-d+\mathrm{2,1}}&0&0\\{dist\_w}_{m-d+\mathrm{3,1}}&{dist\_w}_{m-d+\mathrm{3,2}}&0\end{array}\\ \vdots \\\begin{array}{ccc}{dist\_w}_{m,1}&{dist\_w}_{m,2}&{dist\_w}_{m,3}\end{array}\end{array}&\begin{array}{c}\begin{array}{ccc}0&{dist\_w}_{m-d+1,m-d+2}&{dist\_w}_{m-d+1,m-d+3}\\\dots&0&{dist\_w}_{m-d+2,m-d+3}\\0&\cdots&0\end{array}\\ \vdots \\\begin{array}{ccc}\cdots&{dist\_w}_{m,m-d+1}&0\end{array}\end{array}&\begin{array}{c}\begin{array}{ccc}{dist\_w}_{m-d+1,m-d+4}&\dots&{dist\_w}_{m-d+1,m}\\{dist\_w}_{m-d+2,m-d+4}&\dots&{dist\_w}_{m-d+2,m}\\{dist\_w}_{m-d+3,m-d+4}&\dots&{dist\_w}_{m-d+3,m}\end{array}\\ \vdots \\\begin{array}{ccc}0&\dots&0\end{array}\end{array}\end{array}\right]$$.

###### Definition 3

$\epsilon-d$Neighborhood: For the sample ${a_j} \in D$, considering a sliding window ${L}_{sliding}$ along the fire line starting from the fire line point ${l}_{j}$ corresponds to ${a}_{j}$,${L}_{sliding}=[{l}_{j},{l}_{j+d-1}]$, where $d$ is the size of the sliding window. Let ${D}_{sliding}$ be the sample set of ${L}_{sliding}$, the $\epsilon-d$ neighborhood of ${a}_{j}$ includes all samples in ${D}_{sliding}$, whose weighted distance to ${a}_{j}$ is not greater than $\epsilon$, ${N}_{\epsilon-d}\left({a}_{j}\right)=\{{a}_{i}\in{D}_{sliding}|{dist\_w}_{i,j}({a}_{i},{a}_{j})\le\epsilon\}$.

###### Definition 4

Core object: If the neighborhood of the sample ${a_j}$(${a_j} \in D$) contains at least $MinPts$ samples, that is, $\left| {{N_{\epsilon - d}}\left( {{a_j}} \right)} \right| \geqslant MinPts$, sample ${a}_{j}$ is a core object.

##### SW-DBSCAN algorithm

Based on the continuity and recognizability of forest fire-fighting task area division, the pseudocode of the SW-DBSCAN algorithm is as follows:

Input: Sample set $D=\{{a}_{1},{a}_{2},\ldots, {a}_{m}\}$, parameters $(\epsilon,MinPts,d,\alpha,\beta)$.

Output: Clusters$$\left\{{C}_{k}\right|k=\mathrm{1,2},\ldots, n\}$$.

(1) According to Definition 1 and Definition 2, the weighted distance matrix ${DIST}_{weighted}$ is obtained;

(2) Initialize the set of core objects: ${\Omega}={\varnothing}$

(3) According to Definitions 3 and 4, the core objects of the sample set $D$ are identified and added to ${\Omega}$;

(4) The number of clusters and the set of unvisited samples are initialized: $k=0$, $\varGamma=D$;

(5) When $${\Omega}\ne{\varnothing}$$,

(6) $${\varGamma}_{old}=\varGamma$$

(7) Select a core object $\mathrm{o}\in{\Omega}$ and initialize the queue $Q=<o>$;

(8) $$\varGamma$$=$$\varGamma\backslash\left\{o\right\}$$;

(9) when $$Q\ne{\varnothing}$$,

(10) remove the first sample $q$ in the queue $Q$.

(11) if $${N}_{\epsilon-d}\left(q\right)\ge MinPts$$, then.

(12) add $${N}_{\epsilon-d}\left(q\right)\cap\varGamma$$ to $$Q$$.

(13) $$\varGamma$$=$$\varGamma\backslash\left({N}_{\epsilon-d}\right(q)\cap\varGamma)$$.

(14) $$k=k+1$$, $${C}_{k}$$=$${\varGamma}_{old}\backslash\varGamma$$.

(15) $${\Omega}={\Omega}\backslash{C}_{k}$$

(16) return all clusters$\left\{{C}_{k}\right|k=\mathrm{1,2},\ldots, n\}$.

### Experimental evaluation metrics

To comprehensively evaluate the effectiveness of the SW-DBSCAN algorithm, the following evaluation metrics are defined in the experiments: (1) Running time: the execution time required by the CPU for data clustering in this algorithm; (2) Precision rate: To evaluate the recognizability of forest firefighting task area divisions, let $${n}_{1}$$ be the number of data points clustered as key protected targets (or different fire parts such as fire head, fire flank, and fire tail), and $${n}_{2}$$ be the total number of these key targets in the dataset. The precision is calculated as $$P=\raisebox{1ex}{${n}_{1}$}\!\left/\!\raisebox{-1ex}{${n}_{2}$}\right.$$; (3) Discontinuity rate: to evaluate the continuity clustering effect of forest firefighting task area division, the number of clusters corresponds to discontinuous intervals of the fire line in the clustering results, $${n}_{3}=card\left(A\right)$$, $$A=\left\{{C}_{i}\right|card\left({L}_{{C}_{i}}\right)<max\left({L}_{{C}_{i}}\right)-min\left({L}_{{C}_{i}}\right)+1\}$$, where $${C}_{i}$$ is a certain category after clustering; $${L}_{{C}_{i}}$$ is the subscript set of the fire line point positions corresponding to $${C}_{i}$$, $$i=\mathrm{1,2},3,\ldots, n$$, and $$n$$ is the number of all data clusters. The discontinuity rate is $${P}_{dis}=\raisebox{1ex}{${n}_{3}$}\!\left/\!\raisebox{-1ex}{$n$}\right.$$; (4) Outlier rate: the probability of outliers in clustering results, $${P}_{abn}=\raisebox{1ex}{${n}_{4}$}\!\left/\!\raisebox{-1ex}{$n$}\right.$$, where $${n}_{4}$$ is the number of fireline points labeled as outliers; (5) The commonly used clustering evaluation methods such as the ratio of the mean square distance between clusters to that within clusters (BSS/WSS)^19^, do not consider the continuity and recognizability of forest firefighting task area division. The obtained experimental results are not reasonable. Therefore, based on the evaluation indicators, this study designs the ratio of the continuous weighted mean square distance between clusters to that within clusters, denoted as CW-BSS/WSS. That is, based on the BSS/WSS distance calculation, certain weights are assigned to the dimensions of key protection objects and part sample data. Euclidean distances between the corresponding fire line points in the clustering results are added to the distance calculation between clusters and within clusters. The specific formula is as follows:​​

$$dist({a}_{i},{a}_{j})=\sqrt{{{\upalpha}\bullet({a}_{i}^{ka}-{a}_{j}^{ka})}^{2}+{\upbeta}\bullet{({a}_{i}^{kp}-{a}_{j}^{kp})}^{2}+\sum_{k=2}^{9}{({a}_{i}^{k}-{a}_{j}^{k})}^{2}+{({x}_{{a}_{i}}-{x}_{{a}_{j}})}^{2}+{({y}_{{a}_{i}}-{y}_{{a}_{j}})}^{2}}.$$

where $({x}_{{a}_{i}},{y}_{{a}_{i}})$and $({x}_{{a}_{j}},{y}_{{a}_{j}})$ are the coordinates of the fireline points corresponding to samples ${a}_{i}$ and ${a}_{j}$, respectively.

The shorter the running time, the higher the precision rate, the lower the discontinuity rate and abnormal point rate, and the larger the CW-BSS/WSS value. This indicates that the SW-DBSCAN algorithm has a more accurate clustering effect and higher efficiency. To more accurately evaluate the effectiveness of the algorithm, clustering experiments were conducted on each of the 10 extracted hot lines. The average values of each evaluation metric from the 10 experiments were used as the final experimental results to verify and analyze the accuracy and efficiency of the algorithm.

## Experimental analysis

To validate the effectiveness of the SW-DBSCAN algorithm in forest firefighting task area division, Python 3.7 is used for simulation. The experimental hardware configuration is Intel Core i5-10210Y CPU, Intel UHD Graphics GPU, and Windows 11 operating system.

### Algorithm parameter setting

(1) Discussion on the weights ${\upalpha}$ and ${\upbeta}$.

The SW-DBSCAN algorithm can increase the proportion of key protected objects and parts in distance calculation by introducing weights, thereby achieving the recognizability of forest firefighting task area division. Therefore,${\upalpha}$ and${\upbeta}$ are discussed to determine their impacts on the algorithm. When $({\upalpha},{\upbeta})=\left\{\right(\mathrm{1.5,1.5}),(\mathrm{2,1.5}),(\mathrm{2,2}),(\mathrm{2.5,2}),(\mathrm{2.5,2.5}\left)\right\}$, the clustering effects of the algorithm are compared respectively, as shown in Fig. 3.

As shown in Fig. 3, when ${\upalpha}$ and ${\upbeta}$ increase, the precision rates of key protected objects and fire parts (fire head, fire tail and fire flank) improve significantly. The discontinuity rate decreases, and the outlier rate increases accordingly. This is because when the weight parameters are large, the distance between sample points increases, resulting in a reduction in the number of sample points that meet the neighborhood of core objects. Consequently, the points in the dataset cannot form effective clusters and are mistakenly labeled as outliers. When ${\upalpha}=2.5$ and ${\upbeta}=2.5$, the detection rate stability of discontinuous rate, key protected objects, fire head, fire tail and fire wings is relatively good. The abnormal point rate fluctuates largely, but still in the acceptable range. Therefore, to ensure good recognizability and continuity of the algorithm in forest fire-fighting task area division, and to control the outlier rate in an acceptable range, ${\upalpha}=2.5$ and ${\upbeta}=2.5$ are taken as the default weight parameters.

(2) Discussion on sliding window $d$.

**Fig. 4:**
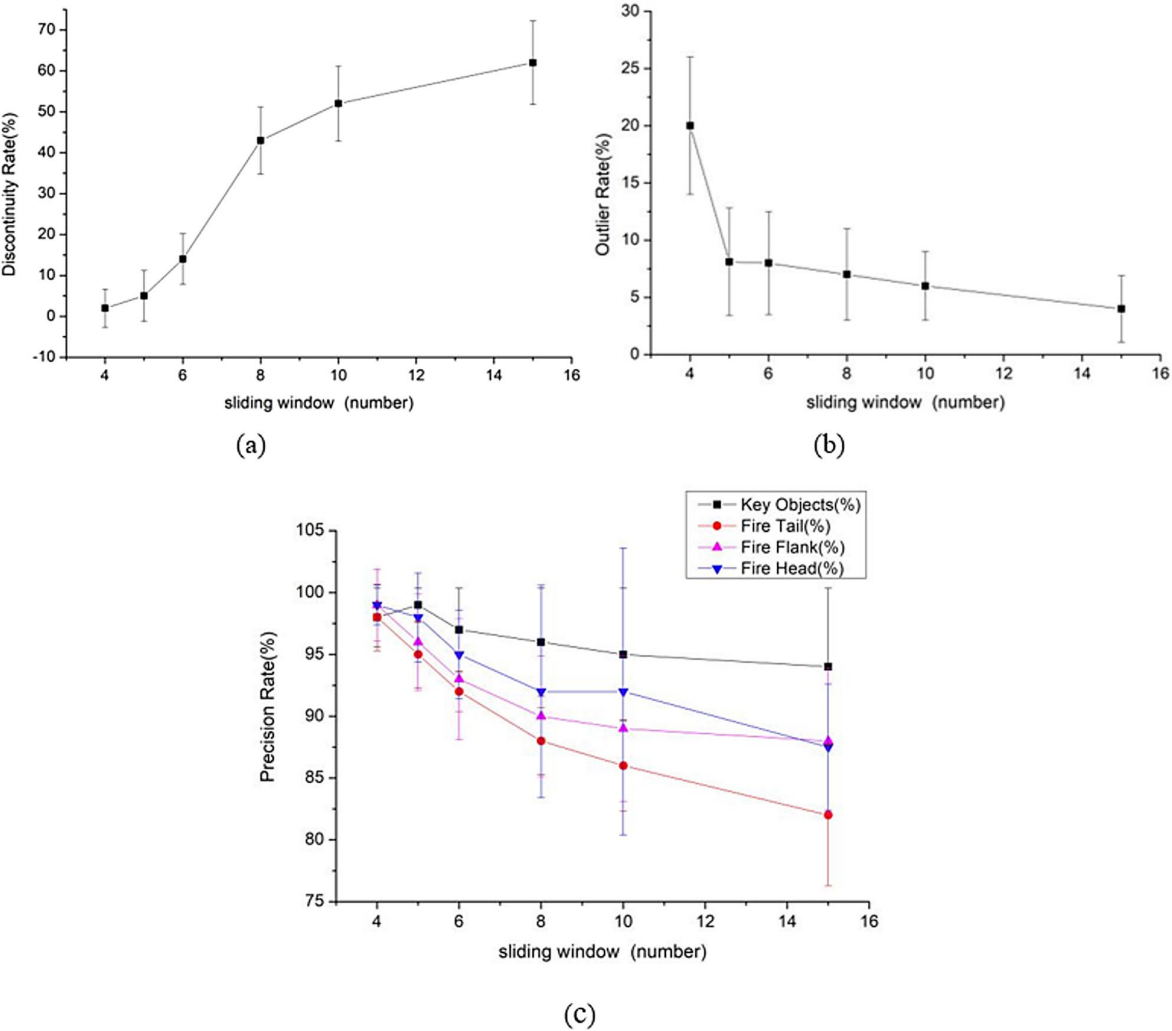
Influence of sliding window $d$ on the algorithm.

According to the continuity requirements of forest firefighting task area division, the sample points in the $\epsilon-d$ neighborhood of a core object should be located around its corresponding fire line point. A sliding window is introduced to reduce the range of the neighborhood $\epsilon-d$. When $d=$4,5,6,8,10 and 15, the clustering effects of the algorithm are compared, as shown in Fig. 4.

As shown in Fig. 4, when $d=$ 4 and 5, the discontinuity rate of the algorithm is low. In addition, the precision rates of the key protected objects, fire head, fire tail, and fire wing are also relatively high with small fluctuations. However, the abnormal point rate of the former is significantly higher than that of the latter. From the error lines, the stability at d = 4 is also worse than that at d = 5. This is because a too small sliding window will cause the sample points in the $\epsilon-d$ neighborhood of the core object to fall outside the corresponding sliding window, making them isolated sample points and mistakenly labeled as outliers. When $d=$6, 8, 10, and 15, the discontinuity rate increases significantly, and the precision rates of identifying key protected objects, the fire head, fire tail and fire wing also decrease gradually. Compared with $d=\mathrm{4,5}$, the stability is also poor, and the incidence of outliers has decreased. Therefore, considering the requirements of continuity and recognizability in forest fire-fighting task area division, the algorithm achieves the best clustering effect when $d=$ 5.

### Sensitivity analysis

(1) Variation of neighborhood radius $\epsilon$.

**Fig. 5:**
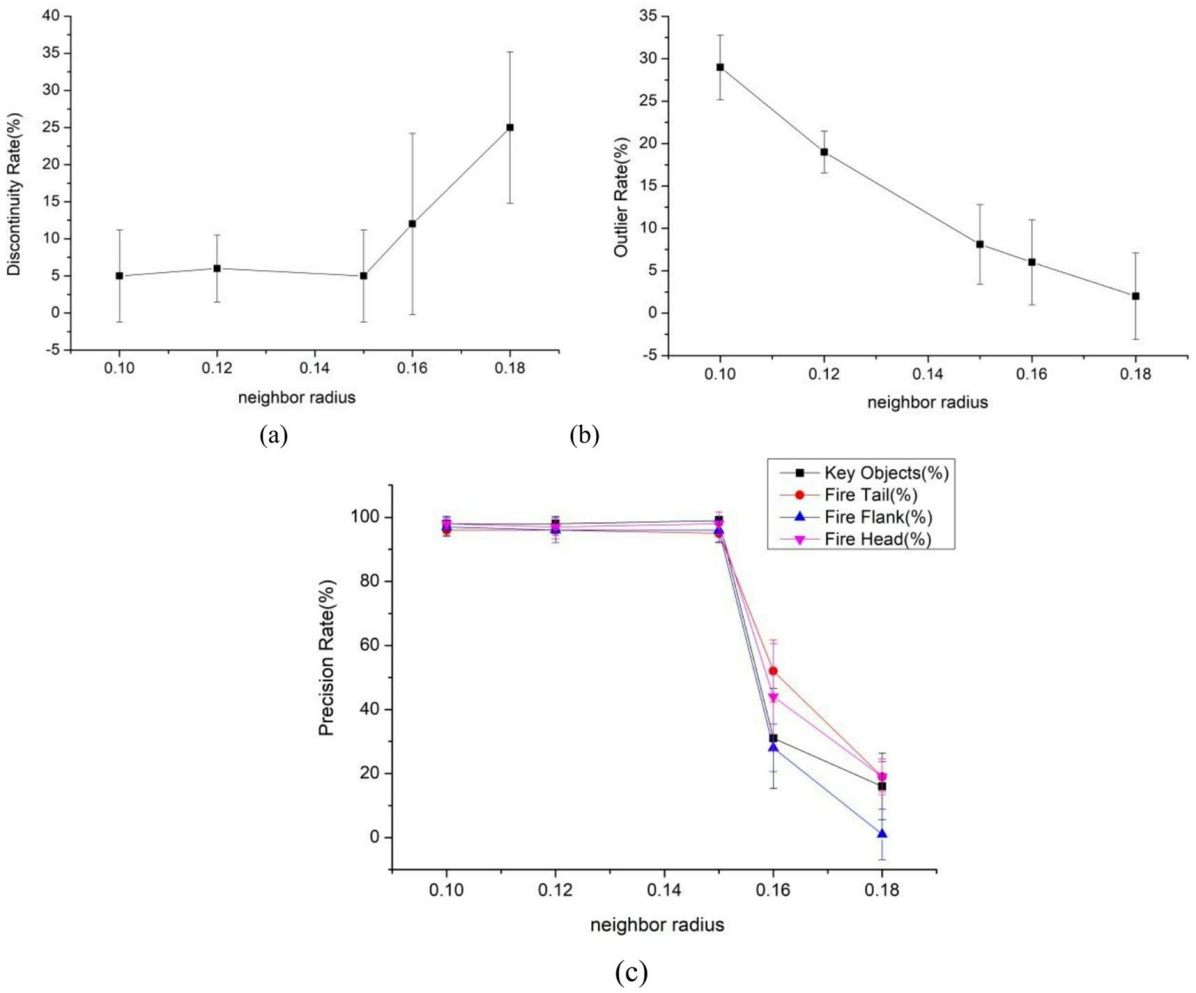
Influence of neighborhood radius ${\epsilon}$ on the algorithm.

The neighborhood radius $\epsilon$ represents the maximum distance threshold of the $\epsilon-d$ neighborhood of a core object, which affects the clustering performance of the algorithm. In this section, the neighborhood radius $\epsilon$ varies. When e = 0.1,0.12,0.15,0.16 and 0.18, the impacts on the outlier rate, discontinuity rate, and precision rates of key protected objects, fire head, fire tail, and fire flank are discussed, as shown in Fig. 5.

As shown in Fig. 5, when $\epsilon=$0.1,0.12,0.15, the value has a small impact on the precision rate of discontinuous rate, key protected objects, fire head, fire tail and fire wing. However, as $\epsilon$ increases, the discontinuous rate of the algorithm increases, and the accuracy rates of key protected objects, as well as the fire head, fire tail and fire wing also decrease, with poor stability. It is believed that when the neighborhood radius is large, the search range of the neighborhood is wide. This increases the probability of finding discontinuous sample points and is prone to merging clusters of different densities, resulting in the misclassification of the key protected objects and samples of different parts into clusters. The ratio of the abnormal point rate to $\epsilon$ is inversely proportional. When $\epsilon=$0.1,0.12, the outlier rate is relatively high. Although the fluctuation of abnormal points in $\epsilon=$0.15 is greater than that in $\epsilon=$0.1, 0.12, the abnormal point rate basically decreases to below 15%, within an acceptable range; when $\epsilon=0.16,0.18$, the anomaly rate significantly decreases. This is because the neighborhood radius $\epsilon$ is too small, resulting in insufficient number of sample points in the $\epsilon-d$ neighborhood of the core object, thereby increasing the number of noise points. Therefore, to ensure the clustering effect of the algorithm, $\epsilon=$0.15 is adopted as the default value of the neighborhood radius $\epsilon$.

(3) Variation of minimum sample number $MinPts$.

**Fig. 6:**
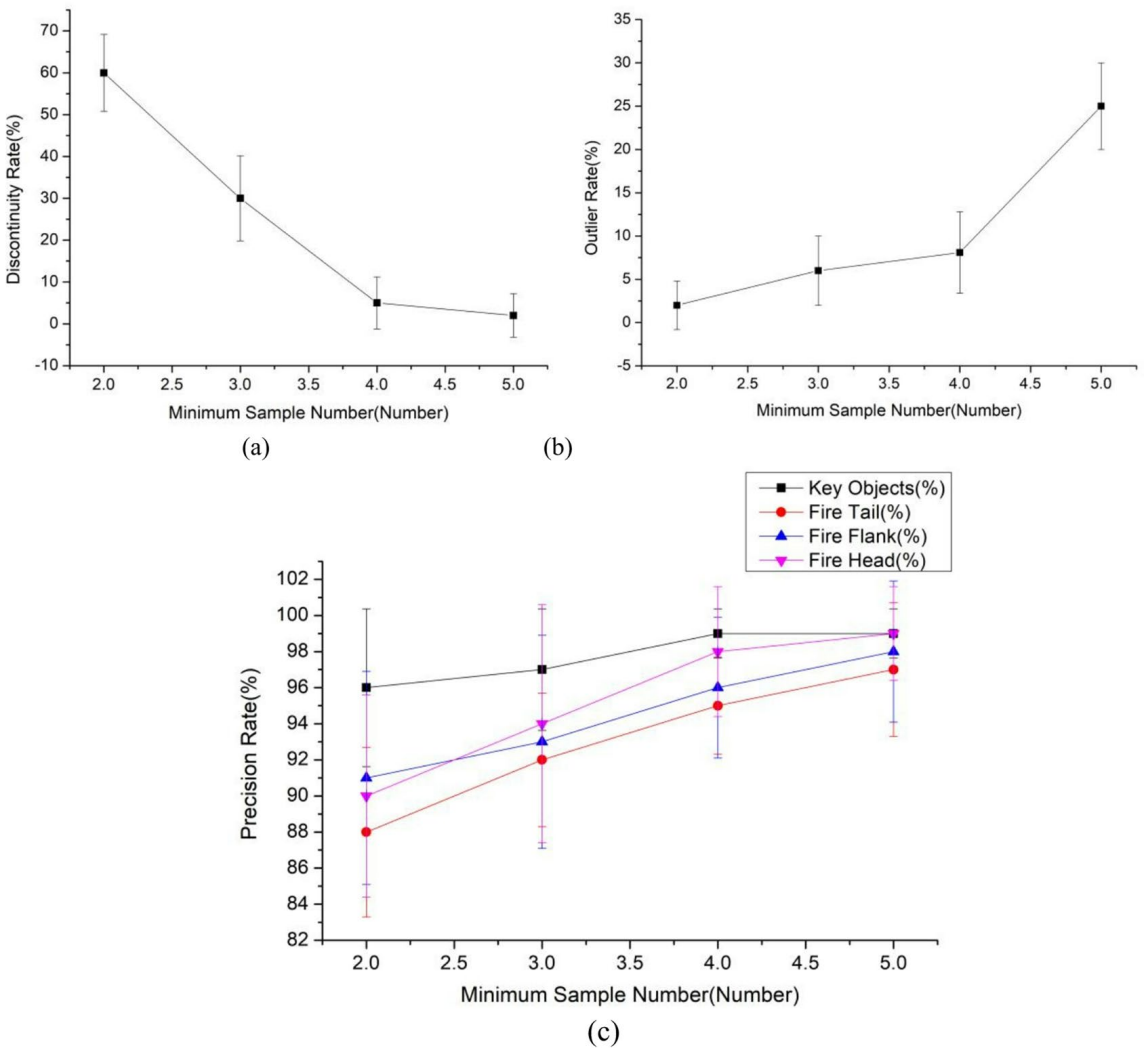
Influence of the minimum sample number $MinPts$ on the algorithm.

The minimum sample number $MinPts$ is the criterion for determining the core objects in the clustering process. This section compares the impacts of $MinPts=$ 2, 3, 4, and 5 on the clustering of the algorithm, as shown in Fig. 6.

As shown in Fig. 6, as $MinPts$ increases, the discontinuity rate significantly decreases, and the stability is improved. However, the rate of abnormal points is opposite, with the rate of $MinPts=4$ significantly lower than that of $MinPts=5$. This is because when the minimum sample size $MinPts$ is large, some low-density clusters are easily overlooked, resulting in insufficient core object quantity and being labeled as abnormal points. The precision rates of key protected objects, fire head, fire tail and fire wing are also improved. $MinPts=4$ is more stable than $MinPts=2,3$. Considering the influence of m on the evaluation indicators of the algorithm, it is believed that $MinPts=4$ can better meet the requirements of the algorithm for continuity and identifiability of the forest firefighting task area division.

### Comparative analysis of SW-DBSCAN algorithm with other spatial clustering algorithms

To achieve the continuity and recognizability of forest fire-fighting task area division, the SW-DBSCAN algorithm introduces a sliding window and weighted distance calculation based on the DBSCAN algorithm to improve the clustering accuracy and efficiency of the algorithm. The SW-DBSCAN algorithm is compared with the DBSCAN algorithm, the OPTICS algorithm^48^, and the HDBSCAN algorithm^49^.

**Fig. 7:**
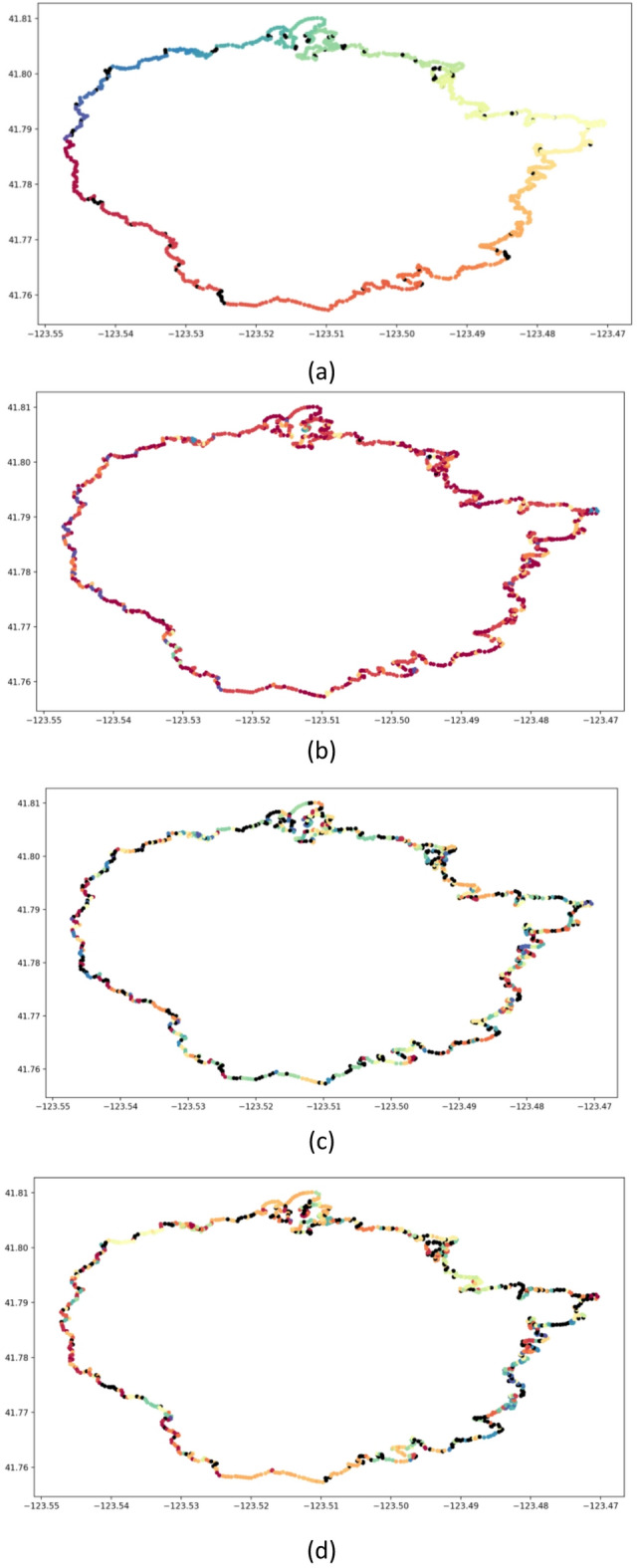
Comparison of clustering effects between SW-DBSCAN algorithm and other spatial clustering algorithms. (**a**) Clustering effect of the SW-DBSCAN algorithm, (**b**) Clustering effect of the DBSCAN algorithm, (**c**) Clustering effect of the OPTICS algorithm, (**d**) Clustering effect of the HDBSCAN algorithm.

The SW-DBSCAN algorithm is configured with the optimal parameters given in 4.2.1 and 4.2.2. Considering that the distance calculation of the DBSCAN algorithm uses Euclidean distance, and the distances between sample points are significantly smaller than those of the SW-DBSCAN algorithm, the algorithm parameters (the neighborhood radius $\epsilon=0.1$ and the minimum sample number $MinPts=4$) are adjusted before comparison to achieve the best clustering effect. The parameter settings of the OPTICS algorithm and the HDBSCAN algorithm are consistent with those of the SW-DBSCAN algorithm. The comparison results are shown in Table 1. The clustering effect of the fire line at a certain moment is shown Fig. 7.

**Table 1:** Comparison of clustering effects between SW-DBSCAN algorithm and other spatial clustering algorithms.

		Discontinuity rate	Outlier rate	Precision rate of key objects	Precision rate of fire tail	Precision rate of fire flank	Precision rate of fire head	CW-BSS/WSS	Running time
SW-DBSCAN Algorithm	Mean	0.05	0.081	0.99	0.95	0.96	0.98	2.432	0.051
	Variance	6.2e−4	4.7e−3	1.3e−4	2.7e−4	3.9e−4	3.6e−4	0.037	1.7e−4
DBSCAN Algorithm	Mean	0.9	0.01	0.28	0.43	0.32	0.3	2.015	0.096
	Variance	4.2e−3	2.5e−5	0.052	0.032	0.114	0.053	0.049	5.4e−4
OPTICS Algorithm	Mean	0.55	0.25	0.99	0.95	0.975	0.93	4.24	2.145
	Variance	4.1e−3	1.8e−3	3.3e−4	5.3e−4	1.3e−4	1.2e−4	0.481	1.4e−3
HDBSCAN Algorithm	Mean	0.62	0.24	0.99	0.94	0.975	0.91	3.51	0.136
	Variance	5.6e−3	1.8e−3	3.3e−4	1.2e−4	1.3e−4	1.6e−4	0.3362	4.4e−4

As shown in Fig. 7, the black dots represent outliers. The SW-DBSCAN algorithm divides the fire line into 106 categories, with good continuity and fewer outliers. The DBSCAN algorithm has 10 clusters, with poor continuity. The OPTICS algorithm and HDBSCAN algorithm have 168 and 122 clusters respectively, with more outliers. As shown in Table 1, it can also be seen that the discontinuity rate of the SW-DBSCAN algorithm is significantly lower than that of the other three methods. However, the outlier rate of the SW-DBSCAN algorithm is lower than that of the DBSCAN algorithm. This is because the DBSCAN algorithm is not limited by the sliding window when searching for sample points near the core object, and can effectively cluster the area of the same density. The precision rate of key protected objects, fire head, fire tail, and fire wing of the SW-DBSCAN algorithm, the OPTICS algorithm and the HDBSCAN algorithm is higher than that of the DBSCAN algorithm, with small variance and better stability. The CW-BSS/WSS value of the SW-DBSCAN algorithm is larger than that of the DBSCAN algorithm, but smaller than that of the OPTICS algorithm and the HDBSCAN algorithm. The stability of the OPTICS algorithm and the HDBSCAN algorithm is poor. The main reason is that the OPTICS algorithm and the HDBSCAN algorithm have a larger number of clusters. Although their continuity is poor, the number of sample points in each class is small, which has a smaller impact on the mean square distance between classes. The mean square distance between points within classes decreases.

The value of CW-BSS/WS is high. The running time of the SW-DBSCAN algorithm is shorter than that of other methods. Compared with other spatial clustering methods, the SW-DBSCAN algorithm can better achieve the continuity and identifiability of the forest firefighting task area division, with higher clustering accuracy and efficiency.

## Conclusions

The SW-DBSCAN algorithm is proposed for forest firefighting task area division. The experimental results show that it has high continuity, recognizability, clustering accuracy, and efficiency. The influence of main parameters of the algorithm on the clustering effect and the comparative analysis between DBSCAN algorithm and SW-DBSCAN algorithm are discussed. The main conclusions are as follows:

1. The recognizability of forest firefighting task area division is mainly affected by the weights ${\upalpha}$ and ${\upbeta}$. The larger the weights, the higher the precision rates of key protected objects and fire parts (fire head, fire tail, and fire flank) on the fire line. The sliding window $d$ can effectively solve the continuity of forest firefighting task area division. The smaller $d$, the lower the discontinuity rate. However, outliers are prone to occur in the clustering results;
2. The neighborhood radius $\epsilon$ determines the range of the$\epsilon-d$ neighborhood of core objects. The minimum sample number $MinPts$ is used to determine whether a sample is a core object. The larger the neighborhood radius $\epsilon$, the higher the discontinuity rate, and the lower the precision rate and outlier rate. Conversely, the change in the minimum sample number $MinPts$ has the opposite effect on the clustering performance;
3. The experimental comparison shows that the SW-DBSCAN algorithm has high precision rates than the DBSCAN for key protection objects and fire parts (fire head, fire tail and fire flank) than, with a significantly lower discontinuity rate. The ratio of the mean square distance within-classes to that between classes is large for continuous weighting and short running time. Although the outlier rate is slightly high, it can be basically maintained below 10% (which is acceptable). In addition, the variance values of all indicators are less than 0.1, indicating that the SW-DBSCAN algorithm can effectively achieve forest firefighting task area division with good stability.

However, there are still some limitations:

First, due to the influence of multiple factors such as technical conditions and organizational management deficiencies, the collection of historical fire site data is incomplete. Future research will focus on improving data collection, parameter optimization, and dynamic task area division;

Second, current study area is limited to the Oak Hill region of Napa County, California, USA. The discussion on algorithm parameters is region-specific. Future verification should be conducted in more complex terrains to further test the effectiveness of parameter settings. Although the manual trial-and-error method is the mainstream for parameter optimization, automatic or adaptive parameter adjustment methods should also be explored;

Third, to simplify the model, the algorithm only considers whether the fire line points are key protected objects. The differences in operational plans for different key protected objects require that the clustering also have identifiability. The algorithm needs to be further improved to identify different key protection objects in forest firefighting task area division. This would provide a clearer and more objective basis for fire suppression force dispatch and the formulation of fire rescue tactics;

Fourth, only the task area division under a single fire line is taken into account, and it is insufficient to cope with the dynamic changes of fires. Future work will focus on the dynamic partitioning method based on the temporal and spatial evolution of the fire line. Through rapid update of partitions, we aim to enhance the real-time situational awareness of rescue personnel, thereby providing key support for optimizing resource allocation and formulating scientific tactical decisions. At the same time, to more clearly present the effect of forest firefighting task division and facilitate rescue, it will be visible on the actual fire map.

## Data Availability

All data supporting the conclusions of this study are available in Zenodo (10.5281/zenodo.18618461), alongside the custom code described in the Code Availability section.

## References

[1] Xu, H. Research on optimization of task scheduling for forest fire rescue helicopter bucket firefighting[J]. *Fire Sci. Technol.*10.20168/j.1009-0029.2024.4.535.6 (2024).

[2] Rodríguez Barreiro, M. & Ginzo Villamayor, M. J. On an optimization model for firefighting helicopter planning. *Int. J. Wildland Fire.* **12**, 1–33. 10.48550/arXiv.2409.07937 (2024).

[3] Dai, J., Chen, W. & Chen, R. Research on task assignment algorithm of heterogeneous aircraft cooperative cluster in dynamic scene. *Comput. Electr. Eng.* **110**, 108781. 10.1016/j.compeleceng.2023.108781 (2023).

[4] Perez-Saura, D. & Fernandez-Cortizas, M. Urban firefighting drones: Precise throwing from UAV. *J. Intell. Robot. Syst.* **108**, 66. 10.1007/s10846-023-01883-6 (2023).

[5] Mugnai, M. & Teppati Losè, M. Towards autonomous firefighting UAVs: Online planners for obstacle avoidance and payload delivery. *J. Intell. Robot. Syst.* **110**, 10. 10.1007/s10846-023-02042-7 (2024).

[6] Umunnakwe, A. & Davis, K. An optimization of UAV-based remote monitoring for improving wildfire response in power systems. *IEEE Open Access J. Power Energy* **10**, 678–688. 10.1109/OAJPE.2023.3337760 (2023).

[7] Xu, W. & Xie, N. Scheduling and route planning for forests rescue: Applications with a novel ant colony optimization algorithm. *Eng. Appl. Artif. Intell.* **155**, 111042. 10.1016/j.engappai.2025.111042 (2025).

[8] Shao, Q. Multi-target firefighting task planning strategy for multiple UAVs under dynamic forest fire environment. *Fire*10.3390/fire8020061 (2025).

[9] Zerveas, G. et al. A transformer-based framework for multivariate time series representation learning[C]//Proceedings of the 27th ACM SIGKDD Conference on Knowledge Discovery & Data Mining. : 2114–2124. (2021). 10.1145/3447548.346740

[10] Zheng, D. Review of multivariate time series clustering algorithms[J]. *J. Front. Comput. Sci. Technol.*10.3778/j.issn.1673-9418.2405013 (2025).

[11] Merhad Ay, L. & Özbakır, S. K. Fixed-centered K-means algorithm[J]. *Exp. Syst. Appl.* **211**, 118656. 10.1016/j.eswa.2022.118656 (2023).

[12] Wang, S. & Yabes, J. G. Hybrid density- and partition-based clustering algorithm for data with mixed-type variables. *J. Data Sci.* **19**(1), 15–36. 10.6339/21-JDS996 (2021).

[13] Tiano, D., Bonifati, A., Ng, R. FeatTS. Feature-based Time Series Clustering[C]//SIGMOD/PODS ‘21: International Conference on Management of Data.0[2025-06-21].10.1145/3448016.3452757

[14] Zhang, L. et al. A novel method for small-target detection in sea clutter: Spectral clustering based on neighborhood density similarity measure. *IEEE Sens. J.* **25**(2), 2988–2997. 10.1109/JSEN.2024.3499954 (2025).

[15] Cai, F. & Feng, J. Hierarchical clustering algorithm based on natural local density peaks. *Signal Image Video Process.* **18**, 7989. 10.1007/s11760-024-03446-0 (2024).

[16] Guan, W. & Wang, P. HC3: A three-way clustering method based on hierarchical clustering. *Cogn. Comput.* **17**, 8. 10.1007/s12559-024-10379-w (2024).

[17] Kim, B., Koo, K., Kim, J. & Moon, B. DISC: Density-based incremental clustering by striding over streaming data[J]. 2021 IEEE 37th International Conference on Data Engineering, 2021,pp. 828 – 839.10.1109/ICDE51399.2021.00077

[18] Zhang, B. & Zhang, L. Image reconstruction of planar electrical capacitance tomography based on DBSCAN and self-adaptive ADMM algorithm. *IEEE Trans. Instrum. Meas.*10.1109/TIM.2023.3284931 (2023).38957474

[19] Zhu, Y.-F. Density-based data clustering algorithm in multi-metric spaces. *J. Softw.* **36**(2), 851–873. 10.13328/j.cnki.jos.007177 (2025).

[20] Kazemi, U. & Soleimani, S. A new approach data processing: Density-based spatial clustering of applications with noise (DBSCAN) clustering using game-theory. *Soft Comput.* **29**(3), 1331–1346. 10.1007/s00500-025-10405-5 (2025).

[21] Abdulnassar, A. A. & Nair, L. R. Performance analysis of Kmeans with modified initial centroid selection algorithms and developed Kmeans9 + model. *Meas. Sensors* **25**, 100666. 10.1016/j.measen.2023.100666 (2023).

[22] Kaduskar, V. et al. Integrating Fuzzy C-Means and DBSCAN: A Hybrid Approach to Medical Data Mining[J].*Fuzzy Inform. Eng.*, **17**(1):108–119. DOI:10.26599/FIE.2025.9270055. (2025).

[23] Punnapathiran, T. & Angsuchotmetee, C. CGNLib: A Python library for Girvan–Newman community detection with customizable node-based centrality metrics. *SoftwareX* **31**, 102193. 10.1016/j.softx.2025.102193 (2025).

[24] Subhajit, S. GVE-Louvain: Fast Louvain algorithm for community detection in shared memory setting[J]. arXiv.2024.DOI:10.48550/arXiv.2312.04876

[25] Huang, L. Crowdsourcing task allocation based on capability hierarchical clustering and role collaboration[J]. Comput. Eng. Des. 2024;45:12. 10.16208/j.issn1000-7024.2024.12.029

[26] Azeddine, E. Neutrosophic logic-based DIANA clustering algorithm[J]. Neutrosophic Sets and Systems.2023(55):498–509. https://fs.unm.edu/nss8/index.php/111/article/view/3205

[27] Weng, S., Fan, Z. & Gou, J. A fast DBSCAN algorithm using a bi-directional HNSW index structure for big data. *Int. J. Mach. Learn. Cybern.* **15**, 3471–3494. 10.1007/s13042-024-02104-8 (2024).

[28] Nicolis, O., Delgado, L., Peralta, B., Díaz, M. & Chiodi, M. Space-time clustering of seismic events in Chile using ST-DBSCAN-EV algorithm. *Environ. Ecol. Stat.* **31**, 509–536. 10.1007/s10651-023-00594-3 (2024).

[29] Jain, P., Bajpai, M. S., Pamula, R. A Modified DBSCAN algorithm for anomaly detection in time-series data with seasonality[J]. Int. Arab J. Inform. Technol. 19(1):23–28. 10.34028/iajit/19/1/3. (2022).

[30] Slimene, M. B. & Ouali, M. S. Anomaly detection method of aircraft system using multivariate time series clustering and classification techniques[J]. IFAC-PapersOnLine 2022;5(10):1582–1587 .10.1016/j.ifacol.2022.09.616

[31] Stehle, F. K. & Vandelli, W. DeepHYDRA: A Hybrid Deep Learning and DBSCAN-Based Approach to Time-Series Anomaly Detection in Dynamically-Configured Systems[J]. ICS ‘24: Proceedings of the 38th ACM International Conference on Supercomputing. 2024:272–285.10.1145/3650200.3656637

[32] Yazhou Ren, N., Wang, M. & Li Z.X. Deep Density-based Image Clustering[J]. 197:105841.DOI: (2020). 10.48550/arXiv.1812.04287

[33] Chen, Z. & Ji, F. Hierarchical clustering algorithm based on Crystallized neighborhood graph for identifying complex structured datasets[J]. *Exp. Syst. Appl.*10.1016/j.eswa.2024.125714 (2025).

[34] Chen, Y., William, R. KNN-DBSCAN: a DBSCAN in high dimensions[J].ACM Transactions on Parallel Computing.2025,**1**(12):1–27 .10.1145/3701624

[35] Erdinç, B., Kaya, M. & Şenol, A. MCMSTStream: Applying minimum spanning tree to KD-tree-based micro-clusters to define arbitrary-shaped clusters in streaming data. *Neural Comput. Appl.* **36**(13), 7025–7042. 10.1007/S00521-024-09443-1 (2024).

[36] Zeng, H., Xuezhong, Q. A parallel DBSCAN algorithm based on KD-tree partitioning and a merging strategy[J],2023 5th International Conference on Machine Learning, Big Data and Business Intelligence (MLBDBI), Hangzhou, China, 2023:195–200. 10.1109/MLBDBI60823.2023.10482269

[37] Ivan, Š & Claudio, K. A parallel algorithm for approximating the silhouette using a ball tree. *J. Parallel Distrib. Comput.* **173**, 115–125. 10.1016/J.JPDC.2022.11.001 (2023).

[38] Cheng, D. & Zhang, C. GB-DBSCAN: A fast granular-ball based DBSCAN clustering algorithm. *Inf. Sci.* **674**, 120731. 10.1016/j.ins.2024.120731 (2024).

[39] Safwan Mohammed, Neeraj, J., Gandhi, C., Bourelly A.D. Euclidean Distance based Adaptive Sampling Algorithm for Disassociating Transient and Oscillatory Components of Signals[J]. *bioRxiv: preprint Serv. biology 2025 DOI* :10.1101/2025.02.23.639754

[40] Fu, J. & Cheng, K. PPA-DBSCAN: Privacy-preserving -approximate density-based clustering. *IEEE Trans. Dependable Secure Comput.*10.1109/TDSC.2024.3375347 (2024).

[41] Ghosh, A., Ghosh, A. K., SahaRay, R. & Sarkar, S. Classification using global and local Mahalanobis distances. *J. Multivar. Anal.* **207**, 105417–105417. 10.1016/J.JMVA.2025.105417 (2025).

[42] Asana, I. M. D. P., Widyantara, I. M. O., Linawati, D. M. & Wiharta Mahalanobis Distance Based DBSCAN For Vessel Near Collision Detection[J]. IEEE 7th International Conference on Information Technology, Information Systems and Electrical Engineering (ICITISEE), 2023:337–342, (2023). 10.1109/ICITISEE58992.2023.10405039

[43] Wang, X., Yue, Y., Zhang, F., Wang, Y. & Zhang, Z. Active detection of interphase faults in distribution networks based on energy relative entropy and Manhattan Distance. *Electr. Power Syst. Res.* **41**, 111397–111397. 10.1016/J.EPSR.2024.111397 (2025).

[44] Zhang, Z. & Chen, J. Exploiting DBSCAN and combination strategy to prioritize the test suite in regression testing. *IET Softw.*10.1049/2024/9942959 (2024).

[45] United States Department of Agriculture, Forest Service, Rocky Mountain Research Station, Missoula Fire Sciences Laboratory. FlamMap: Fire Mapping and Analysis System. FireLab Products: Data and Tools. Accessed July 18, 2024. (2024). https://research.fs.usda.gov/firelab/products/dataandtools/flammap

[46] State Council. Regulations on Forest Fire Prevention. 2008-11-19.

[47] Committee for Examination and approval of Forestry terms. *Forestry Terms* second edition. (Science Press, 2016).

[48] Ankerst, M., Breunig, M. M., Kriegel, H.-P. & Sander, J. OPTICS: Ordering points to identify the clustering structure. *ACM SIGMOD Rec.* **28**, 49–60 (1999).

[49] Campello, R. J. G. B., Moulavi, D. & Sander, J. Density-based clustering based on hierarchical density estimates. Proceedings of the 2009 SIAM International Conference on Data Mining Society for Industrial and Applied Mathematics.pp. 323–333.‌‌ (2013).

